# Respectful Maternal Care Experience in Low- and Middle-Income Countries: A Systematic Review

**DOI:** 10.3390/medicina59101842

**Published:** 2023-10-16

**Authors:** Ayesha Babar Kawish, Muhammad Farooq Umer, Muhammad Arshed, Shahzad Ali Khan, Assad Hafeez, Saman Waqar

**Affiliations:** 1 Alshifa School of Public Health, AlShifa Trust, Rawalpindi 46200, Pakistan; hod-asoph@alshifaeye.org (A.B.K.); saman.naqvi@yahoo.com (S.W.); 2 Department of Preventive Dental Sciences, College of Dentistry, King Faisal University, Hofuf 31982, Saudi Arabia; 3 University Institute of Public Health, Faculty of Allied Health Sciences, University of Lahore, Near Bhuptian Chowk, Lahore 54590, Pakistan; drarshedchaudhary@gmail.com; 4 Office of the Vice Chancellor, Health Services Academy University, Chak Shahzad, Islamabad 44000, Pakistan; shahzad@hsa.edu.pk; 5 Country Representative World Health Organization (WHO), Salmiya 20001, Kuwait; az10@hotmail.com

**Keywords:** respectful maternal care, low- and middle-income countries, maternal mortality, pregnancy, childbirth, evidence-based maternity, maternal health, healthcare systems, prenatal care, continuity of patient care

## Abstract

*Background and Objectives*: Respectful maternity care promotes practices that acknowledge women’s preferences and women and newborns’ needs. It is an individual-centered strategy founded on ethical and human rights principles. The objective of this systematic review is to identify the impact of income on maternal care and respectful maternity care in low- and middle-income countries. *Materials and Methods*: Data were searched from Google Scholar, PubMed, Web of Science, NCBI, CINAHL, National Library of Medicine, ResearchGate, MEDLINE, EMBASE database, Scopus, Cochrane Central Register of Controlled Trials (CENTRAL), and Maternity and Infant Care database. This review followed PRISMA guidelines. The initial search for publications comparing low- and middle-income countries with respectful maternity care yielded 6000 papers, from which 700 were selected. The review articles were further analyzed to ensure they were pertinent to the comparative impact of income on maternal care. A total of 24 articles were included, with preference given to those published from 2010 to 2023 during the last fourteen years. *Results*: Considering this study’s findings, respectful maternity care is a crucial component of high-quality care and human rights. It can be estimated that there is a direct association between income and maternity care in LMICs, and maternity care is substandard compared to high-income countries. Moreover, it is determined that the evidence for medical tools that can enhance respectful maternity care is sparse. *Conclusions*: This review highlights the significance of improving maternal care experiences, emphasizing the importance of promoting respectful practices and addressing disparities in low- and middle-income countries.

## 1. Introduction

Maternity is a term used to define assistance and medical care provided to a woman during her pregnancy and delivery. The state of being expecting or anticipating a child is called pregnancy. Maternity is the time following childbirth [1]. Critical maternal care is a rapidly developing area of clinical practice [1]. The World Health Organization (WHO) recommends respectful maternity care [2], which is defined as components of inclusive and respectful maternity care that include maintaining a woman’s privacy, dignity, and confidentiality; guaranteeing her anonymity from harm and mistreatment; allowing the woman to make an educated decision; and to receive ongoing support throughout her pregnancy and delivery [2]. In providing prospective knowledge and seeking informed consent, continuous access to relatives and local support, women value care that respects their culture, values, and beliefs [3] as well as improving the physical environment and resources, making maternity care better for everyone, providing care that is effective and productive, care stability, and participating in effective conversation [2,3].

The most effective way to maintain safety and ease is through ongoing technological monitoring, maintenance, medication, and to prompt expert assistance when problems emerge [4]. Inclusive and respectful maternity care is a human right, as stated in the 2012 technical guidance on the application of a human rights-based strategy to the implementation of policies and initiatives to decrease preventable maternal morbidity and mortality, as well as the Universal Rights of Childbearing Women Charter from the White Ribbon Alliance [3,5,6]. Access to interventions, medicines, and commodities is insufficient to ensure quality maternal and newborn care [6]. Respectful and inclusive care is a fundamental component of high-quality, clinically secure care, but, more significantly, it feels psychologically and emotionally safe to the woman and her family [7]. This means that treatment must include respect, community knowledge and values, be tailored to women’s needs, and be delivered by health professionals who combine clinical knowledge and skills with interpersonal and cultural competence [8].

Respectful maternity care (RMC) includes all of these aspects and more, and it should be a fundamental component of all maternity care providers in every nation [3,7,8,9]. Another approach is enhanced maternal care [1]. Enhanced maternal care was developed to standardize and deliver the specialized monitoring, treatment, and attention a specific patient may require in maternal or obstetric critical care [1]. The need for skills to treat pregnant, postpartum, or perinatal women who have obstetric, surgical, or medical issues but are not critically ill enough to be admitted to a critical care center drives the development of enhanced maternal care [1]. Any practitioner with the required skills may offer this form of care [1,10].

Moreover, one more approach used for maternal care is evidence-based maternity care. To assist in guiding maternity care decisions and facilitating the best outcomes for mothers and newborns, it uses the most current studies on the effectiveness and safety of specific practices [11]. Evidence-based maternal care in facilities should include humane and dignified care regarding women’s fundamental rights [12]. Respectful maternity care is a term international maternal health organizations use to describe this approach [12]. Obstetric patient treatment is becoming increasingly complex due to comorbidities and advancing maternal age, with socioeconomic factors additionally playing an important part [1]. Another major factor affecting maternity care is income. Evidence demonstrated that lack of income had been associated with poor maternal and newborn health outcomes [13]. Considering the factor of income, maternal care differs in low- and middle-income countries. A list of some low- and middle-income countries is indicated below (Table 1).

Socioeconomic status (SES) is one of the key factors affecting healthcare outcomes. When SES is inadequate, medical care is insufficient, which has been related to detrimental outcomes [14]. Expectant women with low SES may experience more unfavorable pregnancy outcomes [14,15]. Low SES has been linked to pregnancy complications such as preterm birth, abortion, eclampsia, preeclampsia, and gestational diabetes, according to earlier research [14]. Poor prenatal care is related to poor obstetric outcomes, including preterm delivery, preeclampsia, and stillbirth, and women from low SES are less likely to be provided with it [14,15].

Despite improvements in reproductive, maternal, newborn, and pediatric health, disparities in low- and middle-income countries (LMICs) remain significant [13]. In addition to the approximately 4.5 million infants born stillborn or that pass away within the first week of life, it is estimated that nearly 300,000 women worldwide pass away while giving birth each year. Most of these occur in low- and middle-income nations [16] and can be prevented with proper care [13,17]. Giving birth in a health facility lowers maternal and neonatal mortality. However, in low- and middle-income countries, such as Haiti, Kenya, Malawi, Namibia, Nepal, and Tanzania, using facilities more frequently has not always resulted in lower mortality rates. In these nations, many deliveries occur in primary healthcare facilities, where the standard of treatment is substandard [18].

Moreover, in Canada, it has been estimated that every year, approximately 100,000 children are born into poverty [19]. A baby’s health endures greatly during the first few years of life due to poverty and pregnancy, frequently resulting in health inequalities later in life [19]. Low- and middle-income countries carry a significant maternal and newborn mortality burden worldwide. Maternal mortality is typically considered the mother’s death during pregnancy or within the first 42 days following delivery. Maternal mortality rates are 50–100 times higher in low- and some middle-income nations than high-income countries [20]. Most low- and some middle-income countries’ leading causes of maternal mortality are hemorrhage, hypertensive disorders, and maternal infections [20,21,22].

To bring these high levels down, the standard of care must be improved [23]. Maternal and neonatal mortality rates are still high in many low- and middle-income countries, despite substantial decreases over the previous 20 years [18]. Maternal mortality reduction is a top priority for the Sustainable Development Goals (SDGs), which set a global average maternal mortality goal of 70 per 100,000 live births and an additional national target that states that by 2030, no country should have a Maternal Mortality Ratio (MMR) of >140 per 100,000 live births [18]. Additionally, targets were established for each nation’s newborn mortality rate and stillbirth rate to be 12 or less per 1000 live births and 1000 total births, respectively. With an average yearly decrease of 2.9% between 2000 and 2017, there were almost 300,000 maternal and neonatal deaths in women in low- and middle-income countries in 2017 [17].

Advances in maternal and perinatal health and survival were being made through several programs. The worldwide initiatives Every Newborn Action Plan (ENAP) and Ending Preventable Maternal Mortality (EPMM), which focus on stillbirths and neonatal deaths, respectively, seek to hasten and monitor improvements to maternal, perinatal, and newborn health and wellbeing [17]. Although many nations have started the execution process, more aggressive efforts are required to operationalize at the national level to achieve the SDGs [24]. Reviewing current approaches to increase maternal and perinatal survival and wellbeing is necessary, drawing on available data and lessons learned while considering changing epidemiology and demography [17,18,24,25].

According to a study, pregnant women in low-income groups, compared to middle-income, receive worse care, with the most underprivileged being 60% less likely than the least underprivileged to attain antenatal care. The inadequate maternal quality of care constrains improvements in maternal and perinatal results [12]. Poor quality treatment frequently results from a push for births in facilities with insufficient staff, training, infrastructure, and equipment and insufficient evidence-based clinical practice [12]. This type of treatment is known as “Too Little, Too Late” (TLTL). On the other hand, widespread overmedicalization of childbirth has been accompanied by the rapid rise in facility use, especially in middle-income countries (MICs) [26]. The benefits from improvements in maternal and perinatal health may be offset by this excessive medicalization, which we typically refer to as “Too Much, Too Soon” (TMTS) [27]. The clinical care component of the increasing variety and divergence in maternal health is represented by TLTL and TMTS [12]. Individual practitioners in facilities can prevent TLTL or TMTS by adhering to evidence-based clinical standards [12,26,27].

Furthermore, respectful maternity care should be followed by utilizing several measures. Much of the fetal mortality has been decreased by monitoring the fetus during pregnancy and delivery using various methods, such as fetal heart rate monitoring and delivery for signs of distress [20]. The maternal health community has concentrated on ways to lower maternal mortality in low-income and middle-income countries (LMICs), including addressing the direct causes of pregnancy-related deaths, increasing skilled birth attendance, encouraging facility births, and guaranteeing everyone access to essential maternal health care. These approaches have had some degree of effectiveness [12]. A respectful maternity care scale can also assess women’s perceptions of respectful maternity care in health care institutions [28]. The RMC scale of 15 items is a valid and reliable measure. It signifies that hospitals use the RMC scale in urban public health institutions and that other researchers conduct additional exploratory and confirmatory factor analyses [28].

This systematic review aims to determine the impact of income on maternal care and respectful maternity care in low- and middle-income countries.

## 2. Materials and Methods

### 2.1. Search Strategy

In order to execute this review, recent research and review articles/publications based on respectful maternity care in low- and middle-income countries were considered. Data were gathered from the following electronic databases: Google Scholar, PubMed, Web of Science, NCBI, Hindawi, CINAHL, PLoS ONE, National Library of Medicine, ResearchGate, Internal Medicine Journal, MEDLINE, EMBASE database, Science Direct, Scopus, Cochrane Central Register of Controlled Trials (CENTRAL), Maternity and Infant Care (MIC) database, and BioMed.

For this study, we searched through the literature to find articles addressing the meaning of respectful maternity care in low- and middle-income countries and compared them. Studies were selected from years 2010 to 2023 using keywords comprising ‘Respectful maternity’, ‘Care in Low and Middle-Income Countries’, ‘Respectful Maternity Care’, ‘Motherhood’, Childbearing’, ‘Maternity Precautions’, ‘Pregnant Women’, ‘Low and medium income countries’, ‘Attitude of Health Personnel’, ‘Obstetrics’, ‘Delivery’, ‘Delivery Obstetrics’, ‘Infants’, ‘Nursing’, ‘Maternity Care’, ‘Nurse-Patient Relations’, ‘Maternity’, ‘Maternity & care Regulations’, ‘Effects of Income on Pregnancy’, ‘Association between Income and Maternity Care’, ‘Pregnancy and Precautions’, and ‘Income Effect on Maternity Care.’ Search keywords were combined using proximity operators (NEAR, NEXT, WITHIN) and Boolean (AND, OR) operators. Table 2 indicates the data selection strategy for this review.

First, following a search of databases for pertinent articles, text words contained in the title and abstract and index terms used to describe the article were analyzed. Then, across all databases, a second search was conducted using all the discovered keywords, index terms, and MeSH terms for MEDLINE. Third, new studies were found by searching the reference lists of all the studies, reports, and articles. Fourth, databases were searched to identify all related articles and reports in LMICs such as PubMed, Google Scholar, and Google. Titles and abstracts were examined for the search terms. Access was made to the whole texts of the articles that were found. PRISMA guidelines were followed for this review.

The SPIDER framework was employed to determine which studies to include in this systematic review, as shown in Table 3. The PICO model was also used to evaluate databases, as shown in Table 4.

### 2.2. Study Design

* Determine the efficacy of the system in monitoring maternity.
* Efficacy and safety of system advancements in monitoring maternity care.
* Impact of low- and middle-income on maternity care in relative countries.
* Promotion of health and wellness through nutrition, education, and support.
* Maternity care involves decisions about maternal health and helps to support optimal outcomes for mothers and newborns via the highest quality available evidence on the safety and efficacy of particular methods.

### 2.3. Study Outcomes

* Maternity care outcomes could include mortality, live birth, preterm birth, stillbirth, postpartum infection, postpartum hemorrhage, congenital disabilities, and spontaneous and induced abortion.
* Pregnancy or health risk.
* Pregnancy complications and gestational weight gain.
* Safety and efficacy of measures followed during maternity care.

### 2.4. Inclusion Criteria

The following addition and omission criteria were used to filter the titles rather than study relevance. We only selected the studies submitted to peer-reviewed journals for approval that were already published. These studies were taken into consideration to understand the research criteria better.

* All English language research published in peer-reviewed publications was included for review.
* Studies that emphasize the significance of care during the maternity period.
* Studies related to maternity care were included.
* Studies on income impact on maternity were also considered.
* Reviews discussing recent developments in maternity care were focused on.
* Studies between the comparison of low- and middle-income countries and income relation to maternity care included in this review.
* Studies that highlight the precautions during maternity or pregnancy were also considered.
* Studies evaluating the suggestions for care during maternity were also an area of interest.
* The studies included in this systematic review were published in recent years, only between 2010 and 2023.
* Studies about respectful maternity care in low- and middle-income countries were the main focus of gathering useful insight.
* This study included retrospective and prospective randomized studies and analyses, cohort studies, systematic reviews, meta-analyses, case reports, and scoping reviews.

### 2.5. Exclusion Criteria

The exclusion criteria involve

* Other than English language papers were not considered for inclusion.
* Studies focusing solely on the scientific aspects of pregnancy without relevance to maternity care were excluded.
* Papers not aimed at maternity care and advancement in monitoring were excluded from this review.
* The objective was unrelated to maternity care and suggestions to be followed during pregnancy for safer delivery.
* Papers related to maternity care but without a primary objective of assessing the comparison between low- and middle-income countries were excluded from this review.
* Duplicate studies were excluded to avoid the repetition of findings.
* Studies lacking predefined findings’ supporting data.
* Studies whose titles were related to this study but whose text was not relatable were excluded from this review.
* On the other hand, the studies published before 2010 were excluded to ensure recent and up-to-date research.
* It can be stated that studies other than respectful maternity care or countries that do not belong to high- and low-income groups were excluded from this study.

The outcomes of this systematic review were summarized and made explicit by the exclusion and inclusion criteria. Articles that failed to meet the criteria for eligibility were eliminated, duplicate records were eliminated, records with irrelevant titles, abstracts, and keywords were removed, and articles for which no full text was available were also excluded. Most papers were eliminated due to their no direct relevance to this study’s main goal and were written primarily in languages other than English, most commonly Arabic, French, Spanish, and Dutch.

### 2.6. Data Extraction

**Figure 1:**
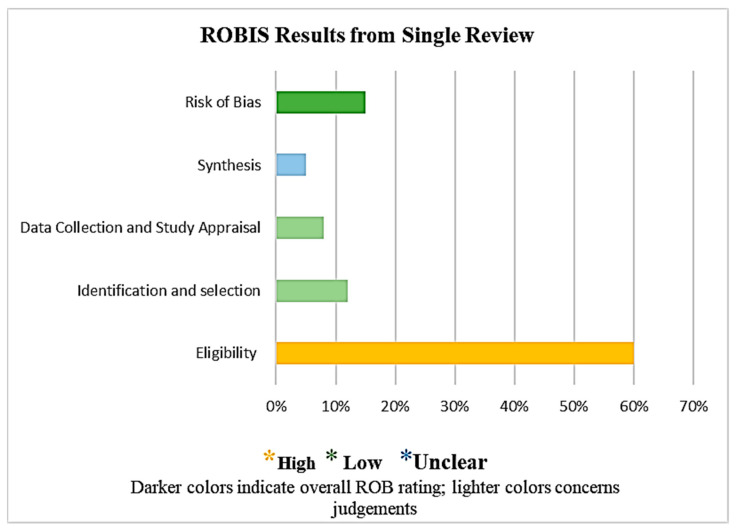
ROBIS results.

Using Microsoft Excel, the researcher extracted and sorted the sample size, study type, duplicates, full-text articles, and empirical studies, making this systematic review approach practicable. The reason for the exclusion and reduction of data is depicted in the flowchart, as seen in Figure 1.

### 2.7. Risk of Bias–Assessment Tool

ROBIS (Risk of Bias in Systematic Reviews) criteria were also implemented to minimize the risk of bias to an extent because it assesses both the risk of bias in a review and (where appropriate) the relevance of a review to the research question. Identifying the scope, reviewing the evidence, holding a face-to-face meeting, and piloting the tool were the four stages comprising the design of ROBIS. Concern levels for each phase 2 domain and the proportion of reviews with high- or low-bias risk range from low to high or were unclear [29]. The use of signaling questions, when combined with a domain-based strategy, stand in line with the most recent techniques to generate the risk of bias tools, as shown in Figure 1. In order to resolve disagreements, the viewpoint of an additional reviewer was sought.

Figure 1 ROBIS findings from a single review are presented in the graphical format as follows: The final section (shaded darker) shows the risk of bias phase assessment. The colored segments show the concerns for each phase 2 ROBIS domain.

Furthermore, the risk assessment was performed by two reviewers based on the study objective, study design, study outcomes, justification by results, study limitations, ethical approval, informed consent from participants, funding, and relevance to this study. The third reviewer’s opinion was also taken to reduce the risk of bias. Risk analysis was performed for the studies included in this review. The reviewer’s risk concerns were low for this study’s objective, low for its relevance to this study, unclear for its limitations, high for funding, unclear for its design and outcomes, and low for its findings, conclusions, ethical approval, and informed consent.

A few concerns were identified with the review process for the phase 2 domain, as shown in the tables below. Concerns of the reviewers range between low, high, and unclear risks, whereas symbols such as Y/PY/PN/N/NI stand for Yes, Probably Yes, Probably No, No, and No information, respectively [29]. (Table 5)

Phase 1. Assessing relevance [30].

Phase 2. Identifying concerns with the review process [30].

**Table 5:** Data collection and study appraisal. Domain 3: describe the procedures used to collect the data, the information that was taken from studies or obtained in other ways, the process used to determine the risk of bias (such as the number of reviewers participating: 02), and the tool that was employed: ROBIS.

	Review 1	Review 2
3.1. Was there any effort made to reduce data gathering errors?	Y	Y
3.2. Were enough research characteristics provided for readers and review authors to interpret the findings?	PY	Y
3.3. Were every relevant study’s findings gathered for the synthesis’s use?	Y	Y
3.4. Was bias risk (or methodological quality) formally evaluated using suitable standards?	Y	Y
3.5. Were measures taken to reduce error in bias risk assessment?	Y	Y
Concerns about the research eligibility criteria’s specification	Low/High/Unclear	
Rationale for concern	Low	

## 3. Results

### Study Selection

**Figure 2:**
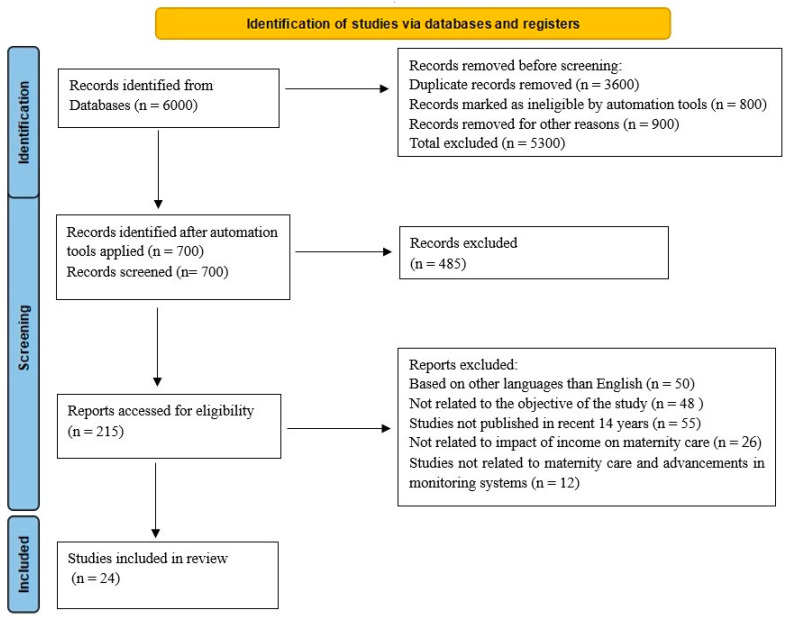
PRISMA, Preferred Reporting Items for Systematic Reviews and Meta-Analyses.

At first, 17,000 results were yielded, upon which search filters such as year of publication, the field of expertise, and article type were applied. The search for publications concerning respectful maternity care in low- and middle-income countries and their comparison or effect of income on maternity care yielded 6000 papers, from which 5300 results were extracted for various reasons, and 700 studies were selected as discussed in Figure 2. However, two relevant articles were separated for future reference. The review articles were further analyzed to ensure they were pertinent to assessing the impact of income on maternity care. Around 215 references were examined for their potential applicability to the medical field of obstetric-led or maternity care. A total of 24 articles were included, with preference given to those published during the last 14 years, from 2010 to 2023. Figure 2 illustrates the PRISMA 2023 article identification flowchart, displaying this systematic review’s many stages in identifying studies.

It comprises the flowchart for the studies that were reviewed. A search of the reviewed literature resulted in 6000 articles, with 5300 duplicates being removed. A total of 700 records were identified after automation tools were used, from which 485 articles were excluded after screening the titles and abstract. There were 215 reports accessed for eligibility. Based on languages other than English, 50 studies were excluded. Moreover, 141 studies were removed for other reasons, leaving 24 eligible studies for inclusion.

**Figure 3:**
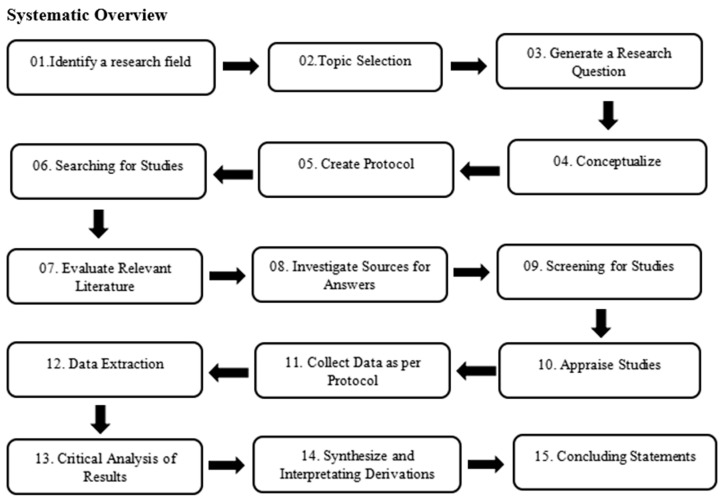
Flow diagram of strategies of systematic overview.

It indicates the strategies involved in systematic review writing. Research study types include systematic reviews. By integrating the findings from all previous scientific studies, a study aims to respond to a particular research question. This offers evidence that is more convincing and reliable than the findings of individual investigations. When performing systematic reviews, the objective is to analyze the thorough, systematic, exact, and clear literature. Additionally, a method based on and modified from Cochrane’s method was employed, as shown in Figure 3. These 15 steps are essential for effectively determining the concept and objective of the research topic. The details of findings from the studies are described as follows:

A total of 81.2% pregnant women received respectful care overall. Age of mothers [AOR = 2.54; 95% CI(1.01–6.43)]; prenatal care follow-ups (AOR = 2.86; 95% CI (1.01–8.20); and maternal occupation (AOR = 5.23; 95% CI (1.15–23.72)). The most important elements of respectful maternity care were found to be conversations with the provider concerning the place of delivery during the antenatal care follow-up [AOR = 5.58; 95% CI: (2.12–14.70)] [31]. From the RMC categories, 76.5% of the women are shielded from physical harm/ill treatment, and 89.2% received fair care devoid of prejudice. The right of women to knowledge, informed consent, and preference protection was upheld in only 39.3% of cases. Birthing at a medical facility (AOR:5.44), discussion of the delivery location (AOR:4.42), daytime delivery (AOR:5.56), longer length of stay (13 h) (AOR:2.10), and delivery time (AOR:2.10). Participation in decision making (AOR: 8.24), obtaining consent prior to the surgery (AOR: 3.45), unplanned pregnancy at the moment (AOR:5.56), three healthcare professionals present during labor (AOR:2.23), and satisfied with the length of time it took to be seen (AOR:2.08) [13]. The continuum of care (CoC) completion rate is low in this study’s site. Only 8.0% of the population had completed CoC. The biggest void, which contributed to the poor CoC, was found between delivery and postnatal care within 48 h after delivery. At six weeks after giving birth, 95% of women had received postnatal care and at least four prenatal visits. A total of 25% of women had postnatal care within 48 h, and 75% had competent assistance with delivery [32]. South Asia shows a decline in service use as women move along the care continuum from pregnancy to childbirth [33]. From “adequate antenatal care” to “adequate delivery care” (0.32) and “adequate child’s immunization” (0.36); from “adequate delivery care” to “adequate postnatal care” (0.78) and “adequate child’s immunization” (0.15)—all along the continuum of care for MNCH—were positively associated and statistically significant at *p* 0.001. The only route association that was adversely associated and significant at *p* 0.001 was the one between “adequate postnatal care” and “adequate child’s immunization” [34]. Despite maternal health and human rights stakeholders’ agreed importance of achieving respectful, non-abusive birth care for all women, there has been a relative lack of formal research on this topic [35]. As natural childbirth ideologies attracted growing North American attention from the mid-1940s, many Canadians sought less-medicalized births [36]. No measure was sufficient to determine women’s experiences of disrespectful and respectful maternity care in low- and middle-income countries. New valid and reliable measures using rigorous approaches to the development of tools are required [37]. A total of 316 of the 321 sampled respondents took part in this study, representing a response rate of 98.4%. Respect and maltreatment were present in 78.2% of cases (95% CI: 73.5–83.2). Unconsented care (86.1%), non-dignified care (37.3%), lack of privacy (33.9%), physical abuse (21.5%), and neglectful care (13.3%) were the most frequent kinds of disrespect and abuse experienced by the mothers. Respect and abuse during facility-based childbirth were strongly correlated with the mothers’ work, an increase in antenatal care visits, and giving birth in a hospital. According to an objective assessment, almost all women (99.7%) experienced D and A during labor. However, only 27.2% of respondents “reported D & A” in terms of their subjective experiences. Facility-based childbirth (OR = 13.49; 10.10–100.16) and lower socioeconomic strata (OR = 2.89; 1.63–5.11) were the primary predictors of reported D and A. In comparison to private health institutions, the chance of reporting D and A was twice as high in public facilities. Women who had previously reported D and A were more likely to choose to give birth somewhere different the second time around (OR = 4.37, 95% CI = 2.41–7.90) [38]. The mean score for respectful maternity care was 62.58, with a range of 15 to 75, while the average score for the entire delivery experience was 3.29, with a range of 1 to 4. A statistically significant direct link between respectful maternity care and a satisfying birthing experience was discovered after accounting for sociodemographic and obstetrical factors (*p* 0.001) [39]. Only 39.4% of women (95% confidence interval: 35.4–43.2) received considerate maternity care, according to this study. Having a high school diploma (adjusted odds ratio 2.47, 95% confidence interval: 1.35–4.50), and receiving follow-up prenatal care adjusted the odds that the pregnancy that was intended (adjusted odds ratio: 3.21, 95% confidence interval: 0.098, 0.03–0.34). Daytime delivery (adjusted odds ratio: 0.47, 95% confidence interval: 0.25–0.89), cesarean section (adjusted odds ratio: 0.69–6.08), and other factors. Respectful maternity care was substantially linked with (adjusted odds ratio: 1.9, 95% confidence interval: 1.33–2.72) [40]. Only 7% women had a negative birth experience. Moreover, factors related to unexpected medical problems were as follows: emergency operative delivery, induction, augmentation of labor, and infant transfer to neonatal care; related to the woman’s social life, such as unwanted pregnancy and lack of support from partner; related to the woman’s feelings during labor, such as pain and lack of control; and related to easier to influence by the caregivers [28]. The proportion of women who had respectful maternity care as a whole was 56.3%. An adjusted odds ratios (AOR) of 2.53 (95% CI: 1.094, 5.867), 2.46 (95% CI: 1.349, 4.482), and 3.092 (95% CI: 1.676, 5.725) for an antenatal care follow-up and above were found to be substantially linked with respectful maternity care [41]. Overall, it is encouraging to see that clinicians treated women with respect and care, yet many of them had unpleasant contacts with them and did not know much about their care. During this study, we saw women being abused verbally and physically. In the unstructured remarks, abandonment and neglect were the forms of disrespect and abuse that were most frequently cited. Except for the Tanzania mainland survey, which had a more evenly distributed mix of facilities with health centers and clinics. Observations were conducted predominantly at hospitals in all countries (80% of deliveries or greater were at hospitals) [42].

## 4. Discussion

### 4.1. Respectful Maternity Care

Respectful maternity care (RMC) is a term that recognizes that safe motherhood needs to extend beyond preventing mortality or morbidity by including respect for women’s fundamental human rights, such as respect for their autonomy, dignity, feelings, choices, and preferences, and such as having a companion as often as possible [43]. The United States Agency for International Development (USAID) adopted a three-pronged strategy of advocacy, study, and support for the implementation in awareness of the significance of this issue. The Maternal and Child Health Integrated Program (MCHIP) has prioritized funding for field-level implementation, while the White Ribbon Alliance and the Translating Research into Action (TRAction) initiative have concentrated on RMC advocacy and research, respectively [33]. In this instance, MCHIP performed the RMC survey to gain more from significant stakeholders about their experiences implementing interventions to promote RMC. A survey about disrespectful treatment and abuse in maternity care, methods for prevention, and strategies for promoting RMC received responses from a convenience group of 48 people from 19 different nations [43].

### 4.2. Respectful Maternity Care Method

The RMC method is individual-focused and founded on moral values and regard for human rights. The White Ribbon Alliance and RMC partners created the Respectful Maternity Care Charter as a reaction to the increasing body of evidence demonstrating disrespect and abuse of childbearing women. It is based on a framework of human rights [43]. Inadequate treatment at all stages has been associated with poor maternal and newborn health outcomes. Disparities in low- and middle-income countries (LMICs) continue to be substantial despite improvements in reproductive, maternal, newborn, and pediatric health [13]. For example, only 14% of women in Sub-Saharan Africa experienced every medical procedure, including at least one antenatal care (ANC), four or more ANC, childbirth with the assistance of a trained birth attendant, a postnatal check (PNC) within 24 h, and family planning assistance within a year of giving birth [13].

### 4.3. Disrespectful and Abusive Childbirth Medical Care

Additionally, throughout the world, women endured disrespectful and abusive childbirth medical care [37]. Consequently, in another study, Freedman et al. 2014 described that respectful and abusive care are interactions or facility conditions that are locally or culturally agreed to be experienced as humiliating or undignified [40]. Physical abuse, non-consent, discrimination, abandonment, and detention in health institutions are all examples of disrespectful and obnoxious treatment in a medical facility [35]. Disrespectful and abusive childbirth care can additionally lead to mental health issues such as childbirth fear, diminished sexuality, post-traumatic stress disorder, and postnatal depression [41]. Even in high-income countries, disrespectful and abusive behavior is not uncommon [36]. However, in middle- to low-income countries (LMICs), where gender inequality is even more severe, it tends to be more prevalent and obvious [44].

### 4.4. Measures to Ensure Respectful Maternity Care in LMIC

The experience of a woman, which is fundamental to RMC, is greatly influenced by the interpersonal interactions with those who are giving medical care or, to put it another way, by a set of behaviors of the providers [45]. The facility’s characteristics and the system the woman interacts with, including the culture, inevitably impact these behaviors [45]. It has been proposed that implementation science frameworks help create behavior change interventions because they can help create a structure for identifying the targets for a program and developing and testing programs that specifically target embedded constructs [45]. However, most RMC interventions do not base their intervention methods on implementation science or behavior change frameworks [38]. Interestingly, most maternal health initiatives do not employ implementation science frameworks, which could aid in the spread of evidence-based practices [45].

### 4.5. Medical Procedures Enhancing RMC

The World Health Organization (WHO) has identified respectful maternity care (RMC) as a fundamental human right since it affects both the mother’s and the child’s health [46,47]. Measures for care or medical procedures that can enhance respectful maternity care include

- Continuous support during labor has been demonstrated to enhance maternal satisfaction, lessen the need for medical interventions, and support a happy birthing experience [39,48].
- Effective pain management provides proper pain management choices, such as epidurals or other pharmacological techniques, can help make labor for women more relaxing and less stressful [40].
- Personalized treatment and communication provides a sense of respect and autonomy can be acquired by adjusting care to each woman’s particular requirements and preferences and including her in decision-making [28]. Respectful maternity care requires effective communication between women and healthcare professionals, including concise and understandable descriptions of alternatives and procedures [49,50].
- Evidence-based and non-invasive approaches enable the risk of unwanted interventions and the potential harm to be decreased by using evidence-based procedures and eliminating unnecessary medical treatments. This includes encouraging physiologically normal childbirth, reducing the frequency of regular episiotomies, and supporting women’s preferences for labor and delivery positions [41].
- Postpartum care and support: RMC and the best outcomes for both mothers and babies depend on comprehensive postpartum care and support, which includes assessing and attending to physical and emotional needs, supporting breastfeeding, and providing information on newborn care [39].
- Formal education, ongoing professional development through mentoring, and clinical job history also help maintain RMC [39].

Finally, it can be summarized that by establishing quality development teams, keeping track of incidents of poor treatment, providing mentorship, and improving staff working conditions, health facilities may establish enabling environments. Health facilities and health systems must be set up to support and respect practitioners and ensure sufficient infrastructure and maternity ward structure to provide respectful care to pregnant women [2]. Following these measures, maternity care can be made respectful in low- and middle-income countries.

### 4.6. Rules of Ethics and Respectful Maternity Care

In order to encourage active participation by professional associations, governments, non-governmental organizations, and civil society in improving quality of care and reducing abuse, neglect, and extortion of childbearing women in facilities, the International Federation of Gynecology and Obstetrics (FIGO) launched the International Childbirth Initiative in cooperation with colleagues from the International Confederation of Midwives, White Ribbon Alliance, the International Paediatrics Association, and the World Health Organization (WHO). Abuse of women, their newborns, and their families, whether physical, verbal, or emotional, is never permitted. Transparent pricing and free or inexpensive healthcare should be provided. Along the whole parenting spectrum, respect each woman’s right to nondiscriminatory, free, or cheap care Respectful maternity care (RMC) is a strategy that places an emphasis on the person, is founded on moral values and respect for human rights, and encourages behaviors that take women’s preferences into account, as well as their needs and those of their unborn children [51,52,53].

### 4.7. Data Analysis

The topics covered in the articles and the developed template aligned with this review’s goal and were used to summarize the general characteristics of the included studies. The template included categories for the title, authors, publishing year, reference number, and conclusions of the included studies. In this repetitive process, literature from the included studies was reviewed, annotated, highlighted, and evaluated. Detailed systematic review is shown in Table 6.

## 5. Conclusions

Concluding this study’s findings, it can be estimated that there is a direct association between income and maternity care. In lower- and middle-income countries, maternity care is substandard compared to high-income countries. Moreover, it is determined that the evidence for medical procedures that can enhance respectful maternity care is sparse. Potential growth of providers’ skills has included transforming attitudes, training on values, and interpersonal communication. Enabling environments can be established within a health facility by establishing quality improvement teams, monitoring poor treatment experiences, mentorship, and improving working circumstances for staff. To provide respectful care, health systems and health facilities must be organized to support and respect clinicians while ensuring sufficient infrastructure and maternity ward organization. Finally, it can be stated that respectful maternity care is a fundamental element of high-quality care and a human right.

## 6. Limitations

This review may have some limitations.

* Although precise criteria were followed to ensure a high-quality review, it is still possible that some research remained unnoticed. This, however, is improbable because the literature was thoroughly examined. Given the review’s brief duration, including a quantitative component might have revealed additional RMC facilitators and impediments.
* We were restricted to RMC-related studies published between 2010 and 2023. Therefore, it is also likely that recent initiatives in other parts of the world have used different methodologies, yielding different findings, and missing older interventions. Our study aimed to determine how a subset of existing procedures may be reframed using this implementation science model and to discuss the possible benefits of doing so, not to be exhaustive.
* Another significant limitation is that we were compelled to base our conclusions on which LMIC countries use certain RMC techniques on data from previously published studies concerning the intervention design. In several instances, minimal information was available because journals had word count restrictions, which would have made it harder for us to allocate domains.
* This article aims to demonstrate how women are treated during pregnancy in lower- and middle-income nations and how cutting-edge techniques can eradicate poor treatment.

## 7. Strengths

* This review appears to have conducted a comprehensive literature search, including multiple databases and employing appropriate search terms. This approach increases the likelihood of capturing relevant studies and minimizing selection bias.
* This review followed a systematic approach, including clear research questions, predefined inclusion/exclusion criteria, and a structured data extraction process. This methodology enhances the rigor and transparency of this review.
* This study focuses on an important and timely topic, highlighting the significance of respectful maternity care. This is an important aspect of maternal healthcare that deserves attention and exploration.

## 8. Research Gap

This review acknowledges the limited evidence and research gaps in interventions to enhance respectful maternity care. By identifying these gaps, this study highlights the need for further research and contributes to advancing knowledge in this area.

## 9. Novelty of Research

The research focuses on respectful maternity care in low-income countries (LMICs) and its implementation, highlighting the limited evidence and understanding of this concept. It identifies gaps, challenges, and potential strategies for promoting respectful maternity care in LMICs. This review emphasizes the need for further research to evaluate interventions, assess effectiveness, and understand their impact on maternal and neonatal health outcomes. This research acknowledges the limited risk of bias in studies and emphasizes the importance of context-specific approaches and methodological rigor in future studies.

## 10. Future Research Directions

Future research directions for respectful maternity care in LMICs include intervention studies, implementation strategies, health system integration, measurement and evaluation, cultural sensitivity, equity and social determinants, and longitudinal studies. These will contribute to the evidence base, inform policy, and improve the provision of respectful maternity care, leading to better maternal and neonatal health outcomes and enhanced patient experiences.

## Figures and Tables

**Table 1:** Low- and middle-income countries.

Low-Income Countries	Middle-Income Countries
Afghanistan	Brazil
Niger	Argentina
Syria	Malaysia
Ugenda	Azerbaijan
Bangladesh	Algeria
South Africa	Ethiopia
Ghana	Tanzania
Iran	Malawi
Burundi	Pakistan
Madagascar	Nepal
Sudan	Indonesia
Central African Republic	India
Liberia	Iran
Somalia	Cambodia
Niger	Bulgaria
Uganda	Cape Verde
Zambia	Egypt
Tajikistan	Sri Lanka

**Table 2:** Relevance of this study. The purpose of ROBIS is to evaluate the risk of bias in reviews by asking questions about interventions, etiology, diagnosis, and prognosis. Name the target question (your overview/guideline question) and the question that the review being evaluated is addressing.

	Reviews Being Assessed
Interventions review	Yes
Aetiology review	Yes
Prognostic review	Unclear
DTA review	No
Does the review’s answer correspond to the target question?	Yes/No/Unclear
Rationale for concern	Yes

**Table 3:** Study eligibility criteria. Domain 1: describe the eligibility requirements for the study, any restrictions on eligibility, and whether there is evidence that the objectives and eligibility requirements were predetermined.

	Review 1	Review 2
1.1. Did the review follow the previously determined objectives and qualifying criteria?	Y	PY
1.2. The eligibility requirements, were they suitable for the review question?	Y	Y
1.3. Were the eligibility conditions clear?	PY	Y
1.4. Were any qualifying criteria constraints based on research features reasonable (e.g., date, sample size, study quality, outcomes measured)?	N	PY
1.5. Were there any constraints in eligibility criteria based on information sources (e.g., publishing status or format, language, data availability)?	PY	Y
Concerns about the research eligibility criteria’s specification	Low/High/Unclear	
Rationale for concern	Low	

**Table 4:** Identification and selection of studies. Domain 2: describe the procedures used to identify and choose studies (such as the number of reviewers involved): 02.

	Review 1	Review 2
2.1. Did a sufficient variety of databases and online sources been used in the search for both published and unpublished reports?	Y	Y
2.2. Were there any techniques employed in addition to database searches to find pertinent reports?	NI	N
2.3. Were the search criteria and structure likely to yield as many relevant studies as possible?	Y	Y
2.4. Were date, publishing format, or language restrictions appropriate?	N	N
2.5. Were measures made to reduce error in study selection?	Y	Y
Concerns about the research eligibility criteria’s specification	Low/High/Unclear	
Rationale for concern	Low	

**Table 6:** List of studies included in this systematic review (2010–2023).

S.No	Title	Author and Year	Sample Size	Country	Study Type	Findings	Limitations	Reference No
01	Respectful maternity care and associated factors among mothers who gave birth in three hospitals of Southwest Ethiopia: A cross-sectional study	Adugna et al., 2023	A total of 348 mothers who gave birth in three hospitals in Southwest Ethiopia	Southwest Ethiopia	Cross-sectional study	A total of 81.2% pregnant women received respectful care overall. Age of the mothers [AOR = 2.54; 95% CI(1.01–6.43)]; prenatal care follow-ups (AOR = 2.86; 95% CI (1.01–8.20); and maternal occupation (AOR = 5.23; 95% CI (1.15–23.72)). The most important elements of respectful maternity care were found to be conversations with the provider concerning the place of delivery during antenatal care follow-up [AOR = 5.58; 95% CI: (2.12–14.70)].	Since the data were gathered in a hospital context, our study may have been influenced by social desirability bias and a fear of reporting abusive care. Another drawback is that some of the ladies were too worn out to reply to several questions because the data were gathered in the early postpartum period.	[31]
02	Respectful maternity care during labor and childbirth and associated factors among women who gave birth at health institutions in the West Shewa zone, Oromia region, Central Ethiopia	Bulto et al., 2020	A total of 567 women	Oromia region, Central Ethiopia	Cross-sectional study	From the RMC categories, 76.5% of the women are shielded from physical harm/ill treatment, and 89.2% received fair care devoid of prejudice. The right of women to knowledge, informed consent, and preference protection was upheld in only 39.3% of cases. Birthing at a medical facility (AOR:5.44), discussion of the delivery location (AOR:4.42), daytime delivery (AOR:5.56), longer length of stay (13 h) (AOR:2.10), and delivery time (AOR:2.10). Participation in decision making (AOR: 8.24), obtaining consent prior to the surgery (AOR: 3.45), unplanned pregnancy at the moment (AOR:5.56), three healthcare professionals present during labor (AOR:2.23), and satisfied with the length of time it took to be seen (AOR:2.08).	Even though the memory bias issue was reduced by performing exit interviews for postpartum mothers right away, the current study is not free of social desirability bias, where some mothers may report the service as having had positive experiences while they are in the medical facilities.	[32]
03	Continuum of care in a maternal, newborn and child health program in Ghana: low completion rate and multiple obstacle factors	Yeji et al., 2015	A total of 1500 mothers with infants	Ghana	Retrospective cross-sectional survey	The continuum of care (CoC) completion rate is low in this study’s site. Only 8.0% of the population had completed CoC. The biggest void, which contributed to the poor CoC, was found between delivery and postnatal care within 48 h after delivery. At six weeks after giving birth, 95% of women had received postnatal care and at least four prenatal visits. A total of 25% of women had postnatal care within 48 h, and 75% had competent assistance with delivery.	This study did not include service availability and other adjustable program factors, which may influence the utilization of MNCH services, such as demand creation efforts, including home visits by CHOs.	[34]
04	Analysis of dropout across the continuum of maternal health care in Tanzania: findings from a cross-sectional household survey	Mohan et al., 2017	A total of 1931 women	Tanzania	Cross-sectional household survey	Dropout from the maternal care continuum was high, especially for the poorest people in rural Tanzania. Only 10% of women reported receiving the ’recommended’ care package (4+ ANC visits, SBA, and 1+ PNC visit), while 1% said they received no care at all. Women’s age (age 20–34 years—OR: 1.77, 95%CI: 1.22–2.56; age 35–49 years—2.03, 1.29–3.2) and awareness of danger indicators (1.75, 1.39–2.1) were also linked favorably with receiving four ANC visits. Women from the fourth (1.66, 1.12–2.47) and highest quintiles of family income (3.4, 2.04–5.66) as well as the top tertile of communities by wealth (2.9, 1.14–7.4) showed a pro-rich bias in facility-based births (a proxy for SBA).	This study is a cross-sectional survey which limits our inference to the associations between independent and outcome variables without the determination of causal direction.	[33]
05	The continuum of care for maternal and newborn health in South Asia: determining the gap and its implications	Alva et al., 2011	Not applicable	Pakistan, Nepal, Bangladesh, India	Review	South Asia shows a decline in service use as women move along the care continuum from pregnancy to childbirth [36]. From “adequate antenatal care” to “adequate delivery care” (0.32) and “adequate child’s immunization” (0.36); from “adequate delivery care” to “adequate postnatal care” (0.78) and “adequate child’s immunization” (0.15)—all along the continuum of care for MNCH—were positively associated and statistically significant at *p* <0.001. The only route association that was adversely associated and significant at *p* <0.001 was the one between “adequate postnatal care” and “adequate child’s immunization”	Only 25–40 percent of women have a postnatal care checkup within 2 days of the child’s birth. The availability of postnatal care soon after birth is also limited among births that did not occur in a health facility.	[35]
06	Associations in the continuum of care for maternal, newborn and child health: a population-based study of 12 sub-Saharan Africa countries	Owili et al., 2016	A total of 137,505 women	A total of 12 Sub-Saharan African Countries	Cross-sectional study	South Asia shows a decline in service use as women move along the care continuum from pregnancy to childbirth [36]. From “adequate antenatal care” to “adequate delivery care” (0.32) and “adequate child’s immunization” (0.36); from “adequate delivery care” to “adequate postnatal care” (0.78) and “adequate child’s immunization” (0.15)—all along the continuum of care for MNCH—were positively associated and statistically significant at *p* <0.001. The only route association that was adversely associated and significant at *p* <0.001 was the one between “adequate postnatal care” and “adequate child’s immunization”.	At the national level, identifying communities that greatly contribute to the overall disparity in health and a well-laid-out follow-up mechanism from pregnancy through to the child’s immunization program which could improve maternal and infant health outcomes and equity.	[36]
07	Enablers and barriers to respectful maternity Care in Low and Middle-Income Countries: a literature review of qualitative research	Mgawadere et al., 2021	Not applicable	A total of 19 low and middle-income countries in Asia and Africa	Review	Respectful maternity care plays a big role in promoting health-seeking behaviors among pregnant women. However, women experience barriers ranging from provider behaviors, work environment, and health system challenges. Ensuring a dignified and respectful working environment could contribute to an increase in health seeking-behaviors and, consequently, a reduction in maternal mortality.	Despite ensuring quality review by following strict criteria, some studies may be missed; however, this is unlikely because of the robust and exhaustive literature search. Considering the short duration of this review, if the quantitative component was added, it may have identified other enablers and barriers to RMC.	[37]
08	Defining disrespect and abuse of women in childbirth: a research, policy and rights agenda	Freedman et al., 2014	Not applicable	Kenya and the United Republic of Tanzania	Cross-sectional study	The growing global movement to promote respectful maternal care has begun strategically using normative standards defined in the laws and policies. However, our projects recognized that simply promoting abstract standards through advocacy and education—or even through legal enforcement and punishment—is unlikely to solve the problem of disrespect and abuse.	Developing interventions to reduce disrespect and abuse, with clearly articulated theories of change and appropriate strategies to assess implementation, will be critical for building an effective global movement for respectful maternal care.	[51]
09	Exploring evidence for disrespect and abuse in facility-based childbirth: report of a landscape analysis	Bowser et al., 2010	Not applicable	Tanzania, Lebanon, Kenya, Brazil, Sierra Leone, Ghana, Zimbabwe, Peru, Burundi, and the United States	Report	Despite maternal health and human rights stakeholders’ agreed importance of achieving respectful, non-abusive birth care for all women, there has been a relative lack of formal research on this topic.	The report reviews many studies from a wide range of countries. The evidence reviewed, however, does not include a validated measurement method for assessing disrespect in facility-based childbirth and does not provide a prevalence estimate.	[52]
10	Encountering abuse in health care; lifetime experiences in a postnatal women-a qualitative study	Schroll et al., 2013	A total of 14 women were selected for an interview	Norway	A qualitative study	Whether AHC is experienced in childhood or adulthood, it can influence women’s lives during pregnancy and childbirth. By recognizing the potential existence of AHC, healthcare professionals have a unique opportunity to support women who have experienced AHC.	However, this study’s participants also revealed potential resources for them to confront, comprehend, and manage their experiences. When addressing future strategies for avoiding AHC, it is important to acknowledge the various forms of dehumanization, focusing on the importance of its opposite: empathy.	[28]
11	Put Right Under Obstetric Violence in Post-war Canada	Wood., 2018	Not applicable	Canada	Review	As natural childbirth ideologies attracted growing North American attention from the mid-1940s, many Canadians sought less-medicalized births.	A historical examination of post-war obstetric practice fundamentally demonstrates that criticisms of modern medicalized birth has its historical roots.	[27]
12	Witnessing obstetric violence during Fieldwork: Notes from Latin America	Castro., 2019	Not applicable	Latin America Countries	Review	Finally, I explain that although reporting on the suffering of women will not, on its own, prevent obstetric violence, increasing its visibility through research can contribute to human rights-based advocacy on behalf of women in labor, to the monitoring of human rights standards, and to the creation of accountability measures within health systems to prevent obstetric violence.	In Proyecto Mujer al Centro (Pregnant Women-Centered Care Project), we are studying the associations among obstetric violence, adverse maternal and child health outcomes, and inequity in the right to health—and, by doing so, we aim to dispel the myth that obstetric violence in a health care setting is uneventful.	[22]
13	A qualitative inquiry of health care workers’ narratives on knowledge and sources of information on principles of Respectful Maternity Care (RMC).	Lusambili et al., 2023	Not applicable	Kenya	Cress-sectional	The Respectful Maternity Care Charter was the subject of a qualitative study that looked at HCWs’ understanding of it and their sources of information in Kenya’s rural Kisii and Kilifi counties. The study’s findings are presented in this publication.	Pre-service medical and nursing curricula and continuing clinical education should include the Respectful Maternity Care Charter. Strategies are required at the policy level to help include respectful maternity care in pre-service training curricula.	[54]
14	Transforming intrapartum care: Respectful maternity care	Bohren et al., 2020	Not applicable	Australia	Review	In order to provide respectful care, health facilities and health systems must be structured in a way that supports and respects providers and ensures adequate infrastructure and organization of the maternity ward.	The provision of respectful care may not be prioritized in the same way as the provision of clinical care. More work is needed to understand how respectful care can be provided, particularly in lower-resource contexts, and how non-recommended practices can be removed from clinical settings.	[2]
15	Quality of measures on respectful and disrespectful maternity care: A systematic review	Dhakal et al., 2021	Not applicable	Not applicable	A systematic review	No measure was sufficient to determine women’s experiences of disrespectful and respectful maternity care in low- and middle-income countries. New valid and reliable measures using rigorous approaches to tool development are required.	Interestingly, although most of the measures included in this evaluation were focused on disrespect and abuse, no measures of disrespectful care could be found.	[45]
16	Magnitude of disrespectful and abusive care among women during facility-based childbirth in Shambu town, Horro Guduru Wollega zone, Ethiopia	Bekele et al., 2020	A total of 321 women	Ethiopia	Cross-sectional study	A total of 316 of the 321 sampled respondents took part in this study, representing a response rate of 98.4%. Respect and maltreatment were present in 78.2% of cases (95% CI: 73.5–83.2). Unconsented care (86.1%), non-dignified care (37.3%), lack of privacy (33.9%), physical abuse (21.5%), and neglectful care (13.3%) were the most frequent kinds of disrespect and abuse experienced by the mothers. Respect and abuse during facility-based childbirth were strongly correlated with the mother’s work, an increase in antenatal care visits, and giving birth in a hospital.	This study had a number of limitations, including the fact that it only looked at women’s subjective experiences, that it was conducted in hospitals where social desirability bias might have been present, and that it was a cross-sectional study, which made it impossible to identify cause-and-effect relationships.	[43]
17	Disrespect and abuse during childbirth in district Gujrat, Pakistan: A quest for respectful maternity care	Azher et al., 2018	A total of 360 women	Pakistan	Cross-sectional study	According to an objective assessment, almost all women (99.7%) experienced D and A during labor. However, only 27.2% of respondents “reported D & A” in terms of their subjective experiences. Facility-based childbirth (OR = 13.49; 10.10–100.16) and lower socioeconomic strata (OR = 2.89; 1.63–5.11) were the primary predictors of reported D and A. In comparison to private health institutions, the chance of reporting D and A was twice as high in public facilities. Women who had previously reported D and A were more likely to choose to give birth somewhere different the second time around (OR = 4.37, 95% CI = 2.41–7.90).	The data used for this study came from women who lived in rural areas; statistics in urban areas can differ slightly. However, given that the women in our sample sought care from both urban and rural health facilities, we anticipate a slight variation. This study’s main weakness was the relatively small percentage of women who gave birth at home and the tiny percentage of them who reported D and A, which led to smaller cell sizes and wider confidence intervals in statistical analysis.	[38]
18	Respectful maternity care and its relationship with childbirth experience in Iranian women: a prospective cohort study	Khadije Hajizadeh., 2020	A total of 334 postpartum women	Tabriz and Iran	Prospective cohort study	The mean score for respectful maternity care was 62.58, with a range of 15 to 75, while the average score for the entire delivery experience was 3.29, with a range of 1 to 4. A statistically significant direct link between respectful maternity care and a satisfying birthing experience was discovered after accounting for sociodemographic and obstetrical factors (*p* 0.001).	Attrition bias and response bias (failure to report occurrences because of feelings of shame and embarrassment or the perception that abusive care is standard care) were two potential biases in this study. We reduced the attrition bias by making timely phone calls (twice a week) to accurately follow-up. The interviews were performed in a private space with participants given assurances of confidentiality and anonymity in order to reduce response bias.	[39]
19	Respectful Maternity Care: Fundamental Human Rights in Labour and Delivery	Adeyemo., 2022	A total of 17 women between the age of 31 and 63	Magu District and Tanzania	Population-based study	The experiences women have with maternal health services reflect a number of variables pertaining to subpar care and violations of many human rights principles. Women identified a variety of methods that the services may provide that would respect human rights principles and acknowledge the existence of subpar treatment. Being respected, receiving the necessary information, and receiving quality medical care were among the major themes.	Emotional support, information gathering, and respectful maternity care prioritize newborns’ rights.	[46]
20	Respectful maternity care and associated factors among mothers who gave birth at public health institutions in South Gondar Zone, Northwest Ethiopia 2021	Ferede et al., 2022	A total of 611women	Ethopia	A multicenter institutional-based cross-sectional study design	Only 39.4% of women (95% confidence interval: 35.4–43.2) received considerate maternity care, according to this study. Having a high school diploma (adjusted odds ratio 2.47, 95% confidence interval: 1.35–4.50), and receiving follow-up prenatal care adjusted the odds that the pregnancy that was intended (adjusted odds ratio: 3.21, 95% confidence interval: 0.098, 0.03–0.34). Daytime delivery (adjusted odds ratio: 0.47, 95% confidence interval: 0.25–0.89), cesarean section (adjusted odds ratio: 0.69–6.08), and other factors. Respectful maternity care was substantially linked with (adjusted odds ratio: 1.9, 95% confidence interval: 1.33–2.72).	It is important to keep in mind the significant limitations of this study when interpreting the results. It was ideal to investigate respectful maternity care using qualitative research and observational data collection methods. The bias toward social desirability can exist. To lessen social desirability bias, each eligible woman was approached in private in a room apart from the maternity unit on the hospital premises.	[40]
21	A negative birth experience: prevalence and risk factors in a national sample	Smith., 2023	A total of 2541 women	Sweden	Longitudinal cohort study	Only 7% women had a negative birth experience. Moreover, factors related to unexpected medical problems were as follows: emergency operative delivery, induction, augmentation of labor, and infant transfer to neonatal care; related to the woman’s social life, such as unwanted pregnancy and lack of support from partner; related to the woman’s feelings during labor, such as pain and lack of control; and related to easier to influence by the caregivers.	Public health initiatives have evaluated nonmedical factors to determine whether they have a broader influence on physical health than traditional medicine, especially in reproductive care.	[44]
22	Magnitude and associated factors of disrespect and abusive care among laboring mothers at public health facilities in Borena District, South Wollo, Ethiopia	Maldie, 2021	A total of 374 women	Ethiopia	Facility-based cross-sectional study	During facility-based deliveries, nearly four out of five (79.4%) women reported at least one sort of disrespect or maltreatment. Non-consented care was the most commonly reported form of disrespect and maltreatment (63.7%). There was a significant association between the wealth index [AOR = 3.27; 95% CI: (1.47, 7.25)], type of health facility [AOR = 1.96; 95% CI: (1.01, 3.78)], presence of companion(s) [AOR = 0.05; 95% CI: (0.02, 0.12)], and presence of complications [AOR = 2.65; 95% CI: (1.17, 5.99)].	The cross-sectional design of this study made it challenging to establish temporal correlations between explanatory variables and the outcome variable, as well as its quantitative design, which was based solely on interviews and excluded other forms of data collecting.	[50]
23	Respectful Maternity Care and Associated Factors Among Women Who Attended Delivery Services in Referral Hospitals in Northwest Amhara, Ethiopia: A Cross-Sectional Study	Yosef et al., 2020	A total of 410 women who gave birth	Northwest Amhara, and Ethiopia	Cross-sectional study	The proportion of women who had respectful maternity care as a whole was 56.3%. Adjusted odds ratios (AOR) of 2.53 (95% CI: 1.094, 5.867), 2.46 (95% CI: 1.349, 4.482), and 3.092 (95% CI: 1.676, 5.725) for the antenatal care follow-up and above were found to be substantially linked with respectful maternity care.	This study had a number of limitations, including the fact that it only examined the subjective experiences of women, the fact that it was carried out in hospitals where social desirability bias was a possibility, and the fact that it was a cross-sectional study, which precluded the identification of cause-and-effect links.	[41]
24	Direct observation of respectful maternity care in five countries: a cross-sectional study of health facilities in East and Southern Africa	Rosen et al., 2015	A total of 2164 women	Ethiopia, Kenya, Madagascar, Rwanda, and the United Republic of Tanzania	Cross-sectional study	Overall, it is encouraging to see that clinicians treated women with respect and care, yet many of them had unpleasant contacts with them and did not know much about their care. During this study, we saw women being abused verbally and physically. In the unstructured remarks, abandonment and neglect were the forms of disrespect and abuse that were most frequently cited. Except for the Tanzania mainland survey, which had a more evenly distributed mix of facilities with health centers and clinics. Observations were conducted predominantly at hospitals in all countries (80% of deliveries or greater were at hospitals). The majority of deliveries that were observed were carried out by female midwives and nurses (87%). A total of 20% of consumers in Ethiopia received medical assistance from doctors, whereas in Madagascar it was just 19%. In 5% of observations, services were provided by nursing and medical students as well as untrained helpers.	The fact that the data collection method was not created, especially to research RMC, constitutes a limitation of this study. Respectful treatment throughout the second and third stage of labor or postpartum was not included on the checklist, and some ideas such as mother detention and consent for operations were completely unaddressed.	[42]

## Data Availability

All of the data are available in the manuscript.

## Abbreviations

World Health Organization (WHO); respectful maternity care (RMC); socioeconomic status (SES); low- and middle-income countries (LMICs); Sustainable Development Goals (SDGs); maternal mortality ratio (MMR); Every Newborn Action Plan (ENAP); Ending Preventable Maternal Mortality (EPMM); Too Little, Too Late (TLTL); middle-income countries (MICs); Too Much, Too Soon (TMTS); Maternal and Child Health Integrated Program (MCHIP); White Ribbon Alliance and the Translating Research into Action (TRAction); antenatal care (ANC); postnatal care (PNC); emergency obstetric care (EmOC); skilled birth attendants (SBA).
